# Hyperuricaemia Prevalence Rates According to Their Physiochemical and Epidemiological Diagnostic Criteria and Their Associations with Cardio-Renal-Metabolic Factors: SIMETAP-HU Study

**DOI:** 10.3390/jcm13164884

**Published:** 2024-08-19

**Authors:** Antonio Ruiz-García, Adalberto Serrano-Cumplido, Ezequiel Arranz-Martínez, Carlos Escobar-Cervantes, Vicente Pallarés-Carratalá

**Affiliations:** 1 Lipids and Cardiovascular Prevention Unit, Pinto University Health Centre, 28320 Madrid, Spain; antoniodoctor@gmail.com; 2 Department of Medicine, European University of Madrid, 28005 Madrid, Spain; 3 Repelega Health Centre, 48920 Portugalete, Spain; adal1953@hotmail.com; 4 San Blas Health Centre, 28981 Madrid, Spain; ezequielarranz@gmail.com; 5 Department of Cardiology, La Paz University Hospital, 28046 Madrid, Spain; carlos.escobar@salud.madrid.org; 6 Department of Medicine, Jaume I University, 12006 Castellon, Spain

**Keywords:** adults, cardiovascular disease, chronic kidney disease, hypertension, hyperuricaemia, prevalence, risk factors, uric acid

## Abstract

**Background:** Scientific societies disagree on serum uric acid (SUA) thresholds for the diagnosis of hyperuricaemia (HU) according to epidemiological or physiochemical criteria (SUA ≥ 7.0 mg/dL for men and ≥6.0 mg/dL for women [HU-7/6]; SUA ≥ 7.0 mg/dL for both genders [HU-7/7], respectively). HU is not included among the diagnostic criteria for metabolic syndrome or cardiovascular-renal-metabolic syndrome (CKM), although it promotes atherosclerosis and is associated with renal and cardiometabolic diseases. Both issues are of utmost importance and need to be clarified, hence the present study aims to assess the prevalence rates of HU and their associations with CKM factors. **Methods:** A cross-sectional observational study was conducted on a random population-based sample of 6489 adults. Bivariate and multivariate analyses were performed on the most well-known renal and cardiometabolic variables of the populations with and without HU-7/7 and HU-7/6. **Results:** The adjusted prevalence rates for HU-7/6 were 13.4% in adult population (18.4% in men; 9.6% in women) and 10.2% (18.4% in men; 3.8% in women) for HU-7/7. The main factors associated independently with HU for both genders were low estimated glomerular filtration rate, hypertension, hypertriglyceridaemia, and alcoholism, regardless of the criteria chosen, as well as albuminuria in women and central obesity in men. **Conclusions:** The prevalence rates of HU increase linearly with age for both genders. The associations of CKM factors with HU diagnosed according to physiochemical criterion are more similar between men and women than those using epidemiological criteria.

## 1. Introduction

International scientific societies disagree on serum uric acid (SUA) thresholds and a single definition for the diagnosis of hyperuricaemia (HU). Some societies [1,2] rely on epidemiological criteria based on the higher average concentrations of SUA in the male population (7.0 mg/dL) than in the female population (6.0 mg/dL) according to an old conference held in Rome in 1961 [1]. Other societies [3,4,5,6] argue that the HU concept should be based on a single pathogenic physiochemical criterion related to the plasma crystallization threshold of monosodium urate (MSU) [3,4,5,6], which occurs without distinction of gender from SUA concentrations around 7 mg/dL [7]. This discrepancy in the diagnosis of HU favours the high variability of the prevalence rates reported for HU and greatly hinders the real assessment of the burden of the disease.

HU is the increase in SUA concentration due to a purine metabolic disorder caused by an imbalance between the increase in uric acid production and the decrease in its elimination, mainly through renal excretion. HU stimulates the production of acute-phase pro-inflammatory molecules that promote the complex process of the pathogenesis of atherosclerosis [8], and it is associated with increased incidence of type 2 diabetes mellitus (DM) [9], metabolic syndrome (MetS) [10], arterial hypertension (HTN) [11,12], cardiovascular diseases (CVD) [13,14,15,16], heart failure (HF) [17], atrial fibrillation (AF) [18], and chronic kidney disease (CKD) [19]. The complexity of the MetS pathogenesis involves different organs and causes predisposition to many diseases. A recent study showed some significant relationships in euthyroid subjects between thyroid volume and function and the results of anthropometric parameters, body composition, and the presence of MetS features [20]. Dysfunction and/or an excess of adipose tissue also promotes the secretion of pro-inflammatory and pro-oxidant molecules that decrease insulin sensitivity, leading to multi-organ damage and promoting the development of multiple clinical disorders and diseases. The consequence of this process is the new so-called cardiovascular–kidney–metabolic (CKM) syndrome, which is defined as a systemic disorder attributable to the pathophysiological interactions between the aforementioned clinical conditions [21].

Cardiometabolic and renal diseases are the most important health disorders worldwide. Their high prevalence rates constitute a public health emergency, not only because the high health care expenditures they cause, but also due to a potential increased risk of premature mortality, a significant clinical implication, and a large burden of cardiovascular disease caused by this excess morbidity [21].

A critically important issue is that HU is not included among the diagnostic criteria for MetS [22] or CKM syndrome [21], despite the fact that it is a disorder that patients with cardiometabolic diseases frequently suffer from, promotes atherosclerosis, and is associated with cardiometabolic diseases.

Both issues are of the utmost importance and need to be clarified. Hence, the present study aims to update and compare the prevalence rates of HU in Spanish adults according to the two aforementioned diagnostic criteria of HU and to evaluate their associations with renal and cardiometabolic factors.

## 2. Materials and Methods

### 2.1. Study Design

This is a multicentre cross-sectional observational sub-study from the SIMETAP study, the design and methodology of which has been reported elsewhere [23], authorised by the Community of Madrid Health Service (Spain) and conducted by 121 physicians from 64 primary healthcare centres. Briefly, all people aged 18 years or older assigned by the Madrid Health Service to research physicians were included for recruitment in a simple random sampling performed by the Excel’s randbetween function. Inclusion criteria: The order indicated by the lists of random numbers was applied to include the study subjects until reaching the sample size necessary to evaluate the objectives of the study. Patients with terminal illnesses or cognitive impairment; institutionalised people; those with dementia, schizophrenia, or moderate or severe psychosis; residents of nursing homes; pregnant women; and people who were participating in other clinical studies were excluded per protocol. Finally, 6489 adults between 18.0 and 102.8 years of age were included in the study, with informed consent and with the necessary clinical and laboratory data to be evaluated (response rate 61.9%). All information assessed in our study was collected from the primary care electronic health records under a real-world data setting.

### 2.2. Definitions of HU, Medical Conditions and Comorbidities

The physiochemical criterion of SUA concentrations determined by the uricase laboratory method ≥ 7.0 mg/dL (416 µmol/L) was considered the primary definition of HU (HU-7/7) for both men and women [3,4,5,6]. Furthermore, we assessed the epidemiological definition (HU-7/6) [1,2] if the SUA concentrations were ≥7.0 mg/dL (HU-7) for men and ≥6.0 mg/dL (357 µmol/L) (HU-6) for women. For both definitions, we also considered HU if the patients were on urate-lowering drug therapy (ULT). The criteria defining CKM syndrome [21] and other comorbidities or medical conditions are reported in Table S1 (Supplementary Materials).

### 2.3. Statistical Analysis

The age- and sex-adjusted prevalence rates were calculated by the direct method according to Spanish population data of the Spanish National Institute of Statistics. Median and interquartile range (IQR) of age of study population were determined. Qualitative variables indicated the number and percentage of each category, using Chi-square test and odds ratios (OR), with a 95% confidence interval (CI). The Shapiro–Wilk test was used to check the data fitting to normal distribution for continuous variables. If the variables showed normal distribution, they were analysed using the arithmetic mean and standard deviation (SD) and compared using Student’s *t*-test or analysis of variance. Cohen’s *d* was used to assess the standardised mean difference, considering the effect size according to the proximity to the following *d*-values: 0.2, small; 0.5, medium; 0.8, large. Bivariate and multivariate analysis were performed in both male and female populations with HU-7 and in the female population with HU-6. To assess the individual effects of comorbidities and clinical conditions on the dependent variables (HU-7/7; HU-7/6), multivariate logistic regression analysis was performed using the backward stepwise method, initially introducing into the model all the variables that showed an association in the bivariate analysis up to a *p*-value < 0.10, except those complex variables with parameters evaluated individually in the analysis. Subsequently, the variable that contributed least to the fit of the analysis was eliminated at each step. All tests were considered statistically significant if the two-tailed *p*-value was <0.05. Statistical analysis was performed using the SPSS for Windows, version 25 (IBM, Armonk, NY, USA).

## 3. Results

### 3.1. General Clinical Characteristics of Overall Study Population

Women represented 56.0% of the study population. Most clinical characteristics were significantly higher in men than in women (Table S2 Supplementary Materials). The median (IQR) ages were 55.0 (42.4–67.5) years in men and 54.5 (41.1–68.8) years in women. The median (IQR) of SUA were 5.6 (4.7–6.6) mg/dL in men and 4.2 (3.5–5.1) mg/dL in women, concentrations very similar to their respective means of SUA, and whose distributions followed Gaussian curves. The mean SUA was 1.31 mg/dL (95% CI: 1.24–1.38) (78 µmol/L) higher in men than in women (*p* < 0.001) (Figure S1 Supplementary Materials).

### 3.2. HU Prevalence Rates

**Figure 1:**
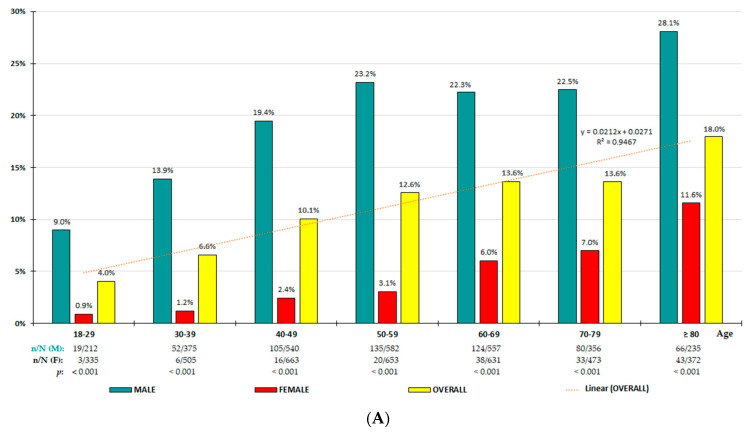

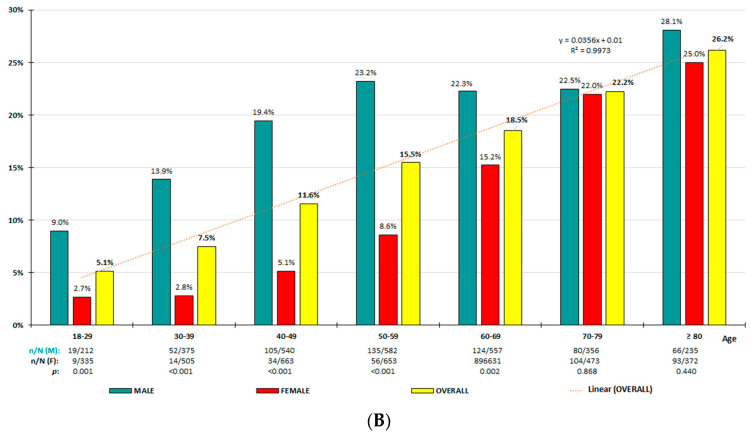
(**A**) Age-specific prevalence rates of HU-7/7; (**B**) Age-specific prevalence rates of HU-7/6. (**A**) HU-7/7: serum uric acid (SUA) ≥ 7.0 mg/dL (416 µmol/L) for both male and female. (**B**) HU-7/6: SUA ≥ 7.0 mg/dL (416 µmol/L) in males and ≥6.0 mg/dL (357 µmol/L) in females. n: number of cases; N: sample size; M: male; F: female; *p*: *p*-value of the difference in percentages (M vs. F).

The crude and adjusted prevalence rates for all study population and according to sex- and age-groups are shown in Table 1. Distributions of age-specific prevalence rates for population with HU-7/7 and for population with HU-7/6 increased quasi-perfectly with age according to linear functions (Figure 1A,B, respectively), being significantly higher in men for all age-groups, except those aged over 70 years for HU-7/6. In patients with HU, the ULT was used in 15.2% of overall population, 20.0% in men, and 13.2% in women (OR 4.2 [95% CI: 2.6–6.8]). Daily doses of ULT ranged between 100 and 300 mg of allopurinol, except one patient who took 100 mg of benzbromarone daily.

### 3.3. Clinical Characteristics of Study Subjects According to Both Diagnostic Criteria

The mean [SD] ages of women with HU-7 (67.5 [15.8] years) and with HU-6 (67.3 [15.4] years) were similarly higher (8.4 and 8.1 years, respectively) and significantly older than those of men (*p* < 0.001). In both male and female populations, most clinical characteristics were significantly higher in HU than non-HU populations. On the contrary, high-density lipoprotein cholesterol and the estimated glomerular filtrate ratio (eGFR) were significantly lower in HU than in non-HU populations. Differences in low-density lipoprotein cholesterol (LDL-C) and aspartate aminotransferase between HU and non-HU populations were non-significant in both male and female populations. Likewise, differences in fasting plasma glucose and glycated haemoglobin A_1c_ were non-significant in the male population, and total cholesterol was non-significant in female populations. According to both physiochemical and epidemiological criteria, the effect sizes according to Cohen’s *d*-values of the standardised mean difference between the HU and non-HU populations were similar among women, and were also larger in women than in men (Table 2).

### 3.4. Effect of CKM Factors and Medical Conditions on HU

**Figure 2:**
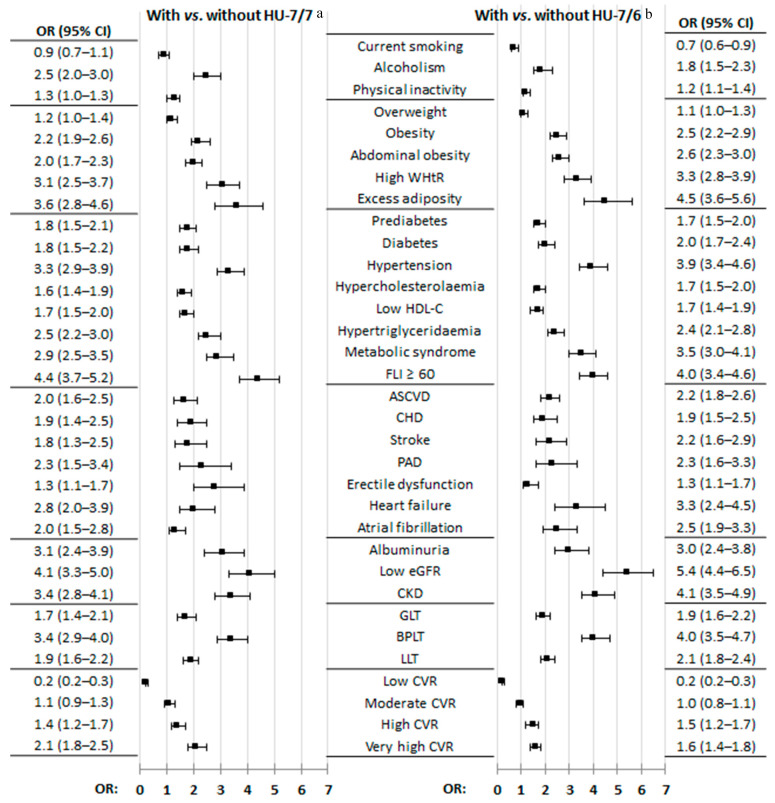
CKM factors and medical conditions in populations with and without hyperuricaemia according to HU-7/7 and HU-7/6 diagnostic criteria. HU-7/7: hyperuricaemia with serum uric acid (SUA) ≥ 7.0 mg/dL (416 µmol/L) for both men and women; HU-7/6: SUA ≥ 7.0 mg/dL (416 µmol/L) for men and ≥6.0 mg/dL (357 µmol/L) for women; No. (%): cases number (percentage); OR: odds ratio; CI: confidence interval; *p*: *p*-value of the difference in percentage; ^a^ N = 683 (with HU-7/7), 5385 (without HU-7/7); ^b^ N = 916 (with HU-7/6), 5152 (without HU-7/6); ASCVD: atherosclerotic cardiovascular disease; BPLT: blood pressure-lowering drug therapy; CHD: coronary heart disease; CKD: chronic kidney disease; CKM: cardiovascular–kidney–metabolic; CUN-BAE: according to its acronym in Spanish, Clínica Universitaria de Navarra—Body Adiposity Estimator; eGFR: estimated glomerular filtration rate; FLI: fatty liver index; GLT: glycaemic-lowering drug therapy; HDL-C: high-density lipoprotein cholesterol; LLT: lipid-lowering drug therapy; PAD: peripheral arterial disease; CVR: cardiovascular risk; WtHR: waist-to-height ratio. The definitions of the CKM factors, comorbidities, and medical conditions are shown in Table S1 (Supplementary Materials).

**Figure 3:**
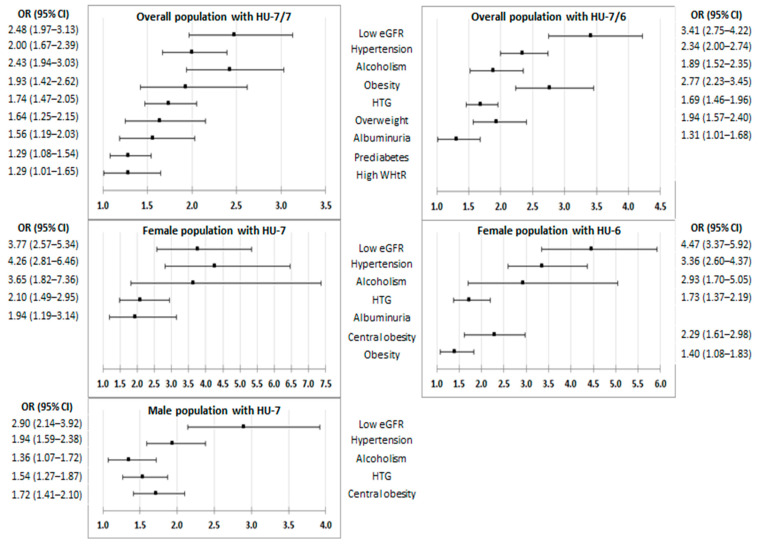
Multivariate analysis results of medical conditions and CKM factors on hyperuricaemia. CI: confidence interval; CKM: cardiovascular–kidney–metabolic; eGFR: estimated glomerular filtration rate; HTG: hypertriglyceridaemia; HU-7/7: hyperuricaemia with serum uric acid (SUA) ≥ 7.0 mg/dL (416 µmol/L) for both men and women; HU-7/6: SUA ≥ 7.0 mg/dL (416 µmol/L) for men and ≥6.0 mg/dL (357 µmol/L) for women; HU-7: SUA ≥ 7.0 mg/dL (416 µmol/L); HU-6: ≥6.0 mg/dL (357 µmol/L); OR: odds ratio. The definitions of the CKM factors, comorbidities, and medical conditions are shown in Table S1 (Supplementary Materials).

**Figure 4:**
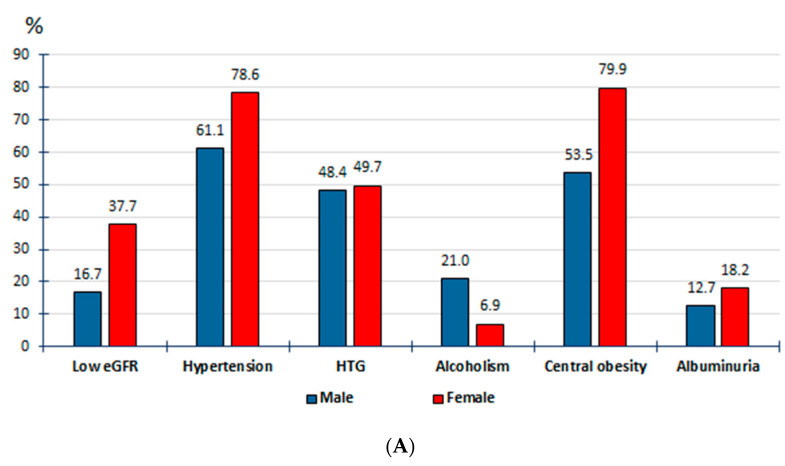

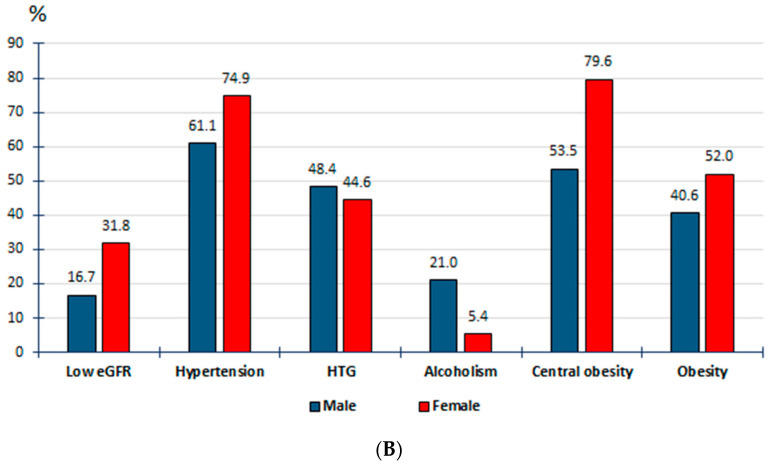
(**A**) Prevalence rates of independent factors associated with HU-7/7; (**B**) prevalence rates of independent factors associated with HU-7/6. HU-7/7: serum uric acid SUA) ≥ 7.0 mg/dL (416 µmol/L) for both sexes. HU-7/6: SUA ≥ 7.0 mg/dL in men and ≥6.0 mg/dL (357 µmol/L) in women. eGFR: estimated glomerular filtration rate; HTG: hypertriglyceridaemia. Definitions of the comorbidities and clinical conditions are shown in Table S1 (Supplementary Materials).

All factors and medical conditions of CKM syndrome were associated with HU according to both criteria, except the variable of physical inactivity according to the physiochemical criterion and overweight according to the epidemiological criteria (Figure 2, Table S3). It is worth mentioning that only 21 patients were on ULT and that they were the same study subjects included in the group diagnosed according to the epidemiological criteria according to the physiochemical criterion. The OR values were very similar when comparing the factors and medical conditions of CKM syndrome, both between the female population with HU-6 and the male population with HU-7 and between both populations with HU-7 (Table S4, Supplementary Materials). Figure 3 shows the graphs of the multivariate analysis of the CKM factors and the medical conditions that are independently associated with HU-7/7 and HU-7/6, both for the general population and according to sex. The most important CKM factors that appear in all the graphs are low eGFR, HTN, and hypertriglyceridaemia (HTG). Likewise, alcoholism appears in all the graphs as a factor independently associated with HU according to both criteria. Figure 4 shows the prevalence rates of the independent factors associated with HU-7/7 and HU-7/6 according to sex, which also include central obesity according to both diagnostic criteria, obesity according to epidemiological criteria, and albuminuria according to the physiochemical criterion.

## 4. Discussion

### 4.1. Hyperuricaemia Prevalence Rates

Average SUA concentrations vary among ethnicities and countries [24], differ by more than 1 mg/dL between men and women, and increase linearly with age in both genders, as we also show in our study.

There is a lack of consensus to define HU, which makes it difficult to compare studies [25]. Some epidemiological prevalence studies define HU as SUA above 6 mg/dL [26,27]. These different criteria may cause the variability in HU prevalence rates to be exaggeratedly high, ranging from 1% in Papua New Guinea to 52% in Taiwan, with variations even in the same countries according to sex or ethnic origin [28]. Other authors [29] and guidelines [1,2] prefer to use two SUA cut-off points to diagnose HU (>7 mg/dL in male; >6 mg/dL in female), arguing epidemiological criteria, because the difference in the HU prevalence rates between the male and female population is too large, as shown in population-based studies carried out in Japan [30]. We consider the mean difference in SUA of 1.0 mg/dL (59 µmol/L) between men and women used for the epidemiological definition of HU to be an easy number to remember, but approximately 25% lower than real-world data, as we report in our study (1.3 mg/dL [78 µmol/L]). Furthermore, the epidemiological cut-off point > 6 mg/dL for females lacks pathogenic relevance for MSU crystals formation. In accordance with the recommendations of clinical guidelines [3,4,5,6], the HU definition should be established in physiochemical terms as the presence of SUA levels above the saturation point for crystallization of MSU (above 6.8 mg/dL) [7], which is unique in humans, regardless of sex. Therefore, herein, we primarily support the definition of HU according to the physiochemical criterion for both genders.

According to the NHANES database from the United States, the prevalence of HU was 20.1% (20.2% in men [with SUA > 7.0 mg/dL]; 20.0% in women [with SUA > 5.7 mg/dL]), or 11.9% (20.2% in male; 4.2% in female) for HU-7/7 [31]. In our study, the HU-7/7 crude prevalence rates were very similar. In a younger Italian population (mean age 43 years), the HU-7/6 prevalence rates were lower (7.3% in men; 2.8% in women) and higher (37.3% and 4.7%, respectively) when 5.6 mg/dL was considered as the cut-off point in both sexes [32]. The HU-7/6 crude prevalence rates of our overall adult population (15.2%) were similar to those detected in the cohort of Spanish patients (mean age 58.7 years) from IBERICAN study [33] (16.3%), although they were higher in men (20.3% vs. 18.7%) and lower in women (11.2% vs. 14.1%), probably due to a slightly lower mean age of our study subjects (55.2 years) and the different sample designs (population-based vs. consecutive recruitment from primary care practices, respectively). The HU-7/6 adjusted prevalence rates of our study were very similar to those found in China (13.5% in adults; 17.3% in males; 10.0% in females) [34].

### 4.2. CKM Factors and Medical Conditions Associated with HU

Body fat distribution is a better risk predictor of comorbidity than obesity determined by waist circumference or body mass index [35]. Furthermore, high WtHR levels are associated with an increased risk of atherosclerotic cardiovascular disease and all-cause mortality [36]. A study carried out in Japan showed that HU was associated with central obesity (WtHR ≥ 0.5), with greater intensity when there was excess weight, but also in the absence of excess weight [37]. We assessed central obesity and adiposity using the WtHR [38] and the CUN-BAE (according to its acronym in Spanish, Clínica Universitaria de Navarra—Body Adiposity Estimator) index [39], respectively, and found that these predictors were more strongly associated with HU (according to both diagnostic criteria) than the obesity, especially in women. Neither overweight nor obesity appeared as independent factors for HU-7 in males in the multivariate analysis. However, central obesity was an independent risk factor for HU-7 in men, and for HU-6 in women. Therefore, central obesity should be identified in HU patients regardless of the existence of excess weight.

The ethanol consumption in alcoholic beverages is the main dietary risk factor contributing to HU, increasing uric acid production and decreasing its renal elimination [40]. Our study showed that alcoholism is an independent risk factor for HU, with a greater association in women than in men for the epidemiological diagnostic criteria, and an even stronger association in females for the physiochemical diagnostic criterion.

Many studies have reported that HU is associated with CVD [13,14,15,16], HF [17], AF [18], and CKD [19], in addition to risk factors such as HTN [11,12], DM [9], MetS [10], HTG, and high LDL-C levels [41]. Our study showed no association between total cholesterol, LDL-C, or peripheral artery disease and HU. No association was found between glycaemic parameters, DM, coronary heart disease, stroke, or AF and male HU, and, on the contrary, these variables did show an association with female HU, probably favoured by the greater age difference with the respective populations without HU (13.0 years in women; 4.9 years in men). HF was associated with HU in both men and women, although it did not appear as an independent risk factor for HU in the multivariate analysis. On the contrary, HTG did appear as an independent risk factor for HU in both genders. 

Some authors reported that HU was an independent risk factor for HTN [11,12] and that this relationship was stronger in females than in males [12]. Our study confirmed that HTN is the second strongest independent risk factor for HU, and that the association between HTN and HU is closer in females than in males for HU-7/6, and even stronger for HU-7/7.

There is a close relationship between HU and MetS [10]. Our study showed a risk of MetS almost three times higher in women than in men. This difference may be due to the greater susceptibility of women to the MetS risk, with lower SUA levels [42]. New insights into pathophysiology of MetS has risen by assessing the involvement of other emerging risk factors such as HU [43]. Our study shows a high prevalence of MetS, CKD, HF, AF, and CVD in patients with HU, which leads us to suspect that CKM syndrome is even more common in them. On the other hand, the metabolic dysfunction-associated steatotic liver disease (SLD) and its possible evolution to more serious liver and cardiovascular diseases is known [44]. In our study, FLI ≥ 60, an accurate predictor of SLD [45], was the factor that was more strongly associated in the female population with HU-7 and HU-6 than in males with HU-7, which could support the close relationship between HU and SLD. Two-thirds of study subjects with HU-7/7 had FLI ≥ 60; therefore, they were more likely to suffer from SLD. Early evaluation of the degree of liver involvement would help in the early application of preventive behaviours and appropriate therapies.

Approximately 70% of SUA is excreted through the kidneys; hence, SUA levels are associated with CKD progression by increasing with the eGFR decrease [19]. Our study also showed stronger associations between HU and CKD, low eGFR, and albuminuria in women than in men according to both criteria, being tighter when the physiochemical criterion was used. Of note, multivariate analysis showed that albuminuria appeared as a factor independently associated with HU in women according to the physiochemical criterion and that it disappeared according to the epidemiological criteria.

HU is a marker that can increase cardiovascular risk (CVR) [46]. However, the optimal value of SUA from which the CKM risk increases has yet to be determined [47]. Our study showed that the percentage of subjects with very high CVR is greater in women than in men, using both the physiochemical and epidemiological criteria. 

It is noteworthy that only 21 patients were on ULT and that they were the same study subjects included in the group diagnosed according to the physiochemical criteria (HU-7/7) as according to the epidemiological criteria (HU-7/6) (Table S4, Supplementary Materials), which would further support the physiochemical criterion because it is closer to the SUA threshold that decides the ULT.

The main limitations of our study were the inability to determine causal relationships or to estimate incidence rates due to cross-sectional design, inter-interviewer variability, possible heterogeneity of the measurement and laboratory equipment, and the HU underdiagnoses due to people being excluded per protocol without SUA data. The values of the variables that were not reported in all study subjects were few, occurred at random, and were similar in the comparison groups, although this could imply a minimal confounding factor in the analysis of the difference between patients with and without HU. Results reporting associations between many factors or clinical conditions and the presence of HU should be interpreted as speculative and with caution, because multiple comparisons using OR and 95% CI could increase the risk of obtaining a statistically significant association by chance (familywise error or alpha-inflation phenomenon). Key strengths include a large sample of people between 18 to 102 years of age, recruited using a population-based method. Assessing the epidemiological magnitude of HU is essential to better plan prevention policies and optimize available health resources. The results reported herein are biologically plausible and consistent with the available scientific information, update the prevalence rates of HU according to the two diagnostic criteria most used by scientific societies, and help to better understand their clinical characteristics and the comorbidities and CKM factors associated with HU.

## 5. Conclusions

The prevalence rates of HU increase linearly with age in both men and women, and the rate higher in men regardless of age group. The associations of CKM factors with HU diagnosed according to the physiochemical criterion are more similar between men and women than those using epidemiological criteria. The results of our study support that that gender should not be a differentiating criterion for the HU diagnosis. A common SUA threshold level of 7 mg/dL for both genders would be more appropriate for HU diagnosis to unify further research on HU. The most important factors associated independently with HU according to the physiochemical criterion are hypertension, low eGFR, hypertriglyceridaemia, and alcoholism in both genders; albuminuria in women; and central obesity in men. Further studies would be needed to address HU as a risk factor for CKM syndrome.

## Figures and Tables

**Table 1:** Hyperuricaemia prevalence rates according to diagnostic criteria and sex and age groups.

		Crude Prevalence Rates				Adjusted Prevalence Rates		
Diagnostic Criteria	Age-Group (Years)	Overall % (95% CI)	Male % (95% CI)	Female % (95% CI)	*p*	Overall (%)	Male (%)	Female (%)
HU-7/7 HU-7/6	<40	5.6 (4.4–6.8) 6.6 (5.3–7.9)	12.1 (9.5–14.7)	1.1 (0.4–1.7) 2.7 (1.6–3.8)	<0.001	5.4 6.4	11.7	1.1 2.7
HU-7/7 HU-7/6	<50	7.6 (6.6–8.7) 8.9 (7.8–10.0)	15.6 (13.5–17.7)	1.7 (1.0–2.3) 3.8 (2.8–4.8)	<0.001	7.1 8.3	14.5	1.6 3.6
HU-7/7 HU-7/6	40–69	12.1 (11.0–13.1) 15.2 (14.0–16.3)	21.7 (19.7–23.7)	3.8 (3.0–4.7) 9.6 (8.3–10.9)	<0.001	11.8 14.7	21.4	3.6 9.0
HU-7/7 HU-7/6	50–69	13.1 (11.7–14.4) 17.0 (15.4–18.5)	22.7 (20.3–25.2)	4.5 (3.4–5.7) 11.8 (10.1–13.6)	<0.001	13.0 16.8	22.8	4.4 11.5
HU-7/7 HU-7/6	≥50	14.0 (12.9–15.1) 19.5 (18.3–20.8)	23.4 (21.4–25.4)	6.3 (5.3–7.3) 16.4 (14.8–18.0)	<0.001	13.9 19.3	23.4	6.2 16.1
HU-7/7 HU-7/6	≥70	15.5 (13.6–17.3) 23.9 (21.2–28.2)	24.7 (21.2–28.2)	9.0 (7.1–10.9) 23.3 (20.5–26.2)	<0.001 0.543	15.5 23.9	24.7	9.1 23.4
HU-7/7 HU-7/6	≥18	11.4 (10.6–12.2) 15.2 (14.3–16.1)	20.3 (18.9–21.8)	4.4 (3.7–5.0) 11.2 (10.2–12.2)	<0.001	10.2 13.4	18.4	3.8 9.6

HU-7/7: hyperuricaemia with serum uric acid (SUA) ≥ 7.0 mg/dL (416 µmol/L) for both male (M) and female (F); HU-7/6: hyperuricaemia with SUA ≥ 7.0 mg/dL (416 µmol/L) in men and ≥6.0 mg/dL (357 µmol/L) in women; CI: confidence interval; *p*: *p*-value of difference in percentages (M vs. F).

**Table 2:** Clinical characteristics of populations with and without hyperuricaemia.

	Male Populations			Female Populations					
	With HU-7 No. 581	Without HU-7 No. 2276	Cohen’s *d* (95% CI)	With HU-7 No. 159	Without HU-7 No. 3473	Cohen’s *d* (95% IC)	With HU-6 No. 406	Without HU-6 No. 3226	Cohen’s *d* (95% CI)
	Mean (SD)	Mean (SD)		Mean (SD)	Mean (SD)		Mean (SD)	Mean (SD)	
Age yr	59.2 (15.5)	54.3 (17.0)	0.3 (0.2; 0.4) ^$$$^	67.5 (15.8)	54.5 (17.9)	0.7 (0.6; 0.9) ^$$$^	67.3 (15.4)	53.5 (17.7)	0.8 (0.7; 0.9) ^$$$^
BMI kg/m^2^	29.5 (4.6)	27.5 (4.4)	0.5 (0.4; 0.5) ^$$$^	31.4 (6.1)	27.0 (5.5)	0.8 (0.6; 1.0) ^$$$^	31.1 (6.0)	26.7 (5.3)	0.8 (0.7; 0.9) ^$$$^
WC cm	102.4 (12.1)	96.9 (12.5)	0.4 (0.4; 0.5) ^$$$^	100.6 (14.1)	89.2 (13.9)	0.8 (0.7; 1.0) ^$$$^	99.6 (14.2)	88.4 (13.6)	0.8 (0.7; 0.9) ^$$$^
WtHR	0.60 (0.07)	0.57 (0.08)	0.4 (0.3; 0.5) ^$$$^	0.65 (0.09)	0.56 (0.10)	0.9 (0.7; 1.1) ^$$$^	0.64 (0.09)	0.56 (0.09)	0.9 (0.8; 1.0) ^$$$^
CUN-BAE adiposity	31.3 (5.6)	28.3 (6.3)	0.5 (0.4; 0.6) ^$$$^	45.3 (5.9)	39.0 (7.4)	0.9 (0.7; 1.0) ^$$$^	45.0 (5.9)	38.6 (7.4)	0.9 (0.8; 1.0) ^$$$^
SBP mmHg	126.8 (15.0)	123.4 (13.7)	0.2 (0.2; 0.3) ^$$$^	129.3 (16.2)	119.8 (16.1)	0.6 (0.4; 0.8) ^$$$^	129.5 (15.6)	119.1 (15.9)	0.7 (0.6; 0.8) ^$$$^
DBP mmHg	76.3 (10.0)	74.5 (9.2)	0.2 (0.1; 0.3) ^$$$^	75.5 (9.4)	72.0 (9.9)	0.4 (0.2; 0.5) ^$$$^	75.7 (9.6)	71.7 (9.8)	0.4 (0.3; 0.5) ^$$$^
FPG mg/dL ^a^	100.3 (22.6)	99.5 (29.6)	0.0 (−0.1; 0.1) ^NS^	101.0 (24.0)	92.7 (23.2)	0.4 (0.2; 0.5) ^$$$^	101.3 (25.3)	92.0 (22.8)	0.4 (0.3; 0.5) ^$$$^
HbA_1c_ % ^b #^	5.74 (0.75)	5.70 (0.97)	0.0 (−0.1; 0.1) ^NS^	5.99 (0.93)	5.55 (0.85)	0.5 (0.4; 0.7) ^$$$^	5.95 (0.93)	5.52 (0.83)	0.5 (0.4; 0.6) ^$$$^
TC mg/dL ^c^	191.2 (39.9)	187.5 (38.8)	0.1 (0.0; 0.2) ^$^	199.8 (42.2)	196.3 (39.1)	0.1 (−0.1; 0.3) ^NS^	198.8 (42.1)	196.1 (38.9)	0.1 (0.0; 0.2) ^NS^
HDL-C mg/dL ^c^	47.1 (12.0)	49.8 (12.7)	−0.2 (−0.3; −0.1) ^$$$^	53.8 (13.4)	59.5 (14.7)	−0.4 (−0.6; −0.2) ^$$$^	56.0 (14.2)	59.7 (14.7)	−0.3 (−0.4; −0.2) ^$$$^
Non-HDL-C mg/dL ^c^	144.1 (39.1)	137.7 (38.7)	0.2 (0.1; 0.3) ^$$$^	146.0 (38.4)	136.7 (38.1)	0.2 (0.1; 0.4) ^$$^	142.9 (40.5)	136.4 (37.8)	0.2 (0.1; 0.3) ^$$^
TG mg/dL ^d^	162.3 (132.0)	129.1 (90.5)	0.3 (0.2; 0.4) ^$$$^	149.8 (108.5)	106.6 (60.6)	0.7 (0.5; 0.8)	144.6 (93.2)	103.9 (57.8)	0.7 (0.6; 0.8) ^$$$^
SUA mg/dL ^e^	7.61 (1.16)	5.21 (1.02)	2.0 (1.9; 2.1) ^$$$^	7.52 (1.02)	4.25 (1.05)	3.1 (3.0; 3.3) ^$$$^	6.80 (0.89)	4.09 (0.91)	3.0 (2.9; 3.1) ^$$$^
Creatinine mg/dL ^f^	1.07 (0.38)	0.93 (0.26)	0.5 (0.4; 0.6) ^$$$^	0.99 (0.52)	0.7 (0.23)	1.0 (0.8; 1.2) ^$$$^	0.92 (0.48)	0.73 (0.21)	0.8 (0.7; 0.9) ^$$$^
eGFR mL/min/1.73 m^2^	80.7 (21.6)	92.0 (18.5)	−0.6 (−0.7; −0.5) ^$$$^	68.8 (25.1)	92.2 (20.3)	−1.1 (−1.3; −1.0) ^$$$^	72.5 (24.3)	93.5 (19.4)	−1.1 (−1.2; −1.0) ^$$$^
uACR mg/g ^g^	34.1 (111.3)	17.3 (69.2)	0.2 (0.1; 0.3) ^$$$^	33.7 (81.6)	12.2 (36.1)	0.6 (0.4; 0.7) ^$$$^	25.4 (70.1)	11.6 (33.3)	0.4 (0.3; 0.5) ^$$$^

HU-7 (hyperuricaemia): serum uric acid (SUA) ≥ 7.0 mg/dL (416 µmol/L). HU-6: SUA ≥ 6.0 mg/dL (357 µmol/L). SD: standard deviation; Cohen’s *d*; standardised mean difference (effect size according to *d*-values: 0.2 small; 0.5 medium; 0.8 large); CI: confidence interval; ^$$$^*: p*-value of the difference in means < 0.001; ^$$^*: p* < 0.01; ^$^*: p* < 0.05; ^NS^: non-significant. The definitions of the variables are shown in Table S1 (Supplementary Materials). BMI: body mass index; CUN-BAE-adiposity: adiposity or body fat index CUN-BAE (according to its acronym in Spanish, Clínica Universitaria de Navarra—Body Adiposity Estimator); DBP: diastolic blood pressure; eGFR: estimated glomerular filtration rate; HbA_1c_: glycated haemoglobin A_1c_ (^#^ No. with vs. without HU-7 [men]: 482 vs. 1845; No. with vs. without HU-7 [women]: 132 vs. 2719; No. with vs. without HU-6 [women]: 347 vs. 2504); HDL-C: high-density lipoprotein cholesterol; SBP: systolic blood pressure; TC: total cholesterol; TG: triglycerides; uACR: urine albumin–creatinine ratio; WC: waist circumference; WtHR: waist-to-height ratio. ^a^ To convert from mg/dL to mmol/L, multiply by 0.05556; ^b^ to convert from % (DCCT) to mmol/mol (IFCC), subtract 2.15 and multiply by 10.929; ^c^ to convert from mg/dL to mmol/L, multiply by 0.02586; ^d^ to convert from mg/dL to mmol/L, multiply by 0.01129; ^e^ to convert from mg/dL to mmol/L, multiply by 0.05948; ^f^ to convert from mg/dL to mmol/L, multiply by 0.08842; ^g^ to convert from mg/g to mg/mmol, multiply by 0.01131.

## Data Availability

After deliberation, the data presented in this study are available upon request from the corresponding author. Data availability is restricted to participating researchers and, therefore, unavailable to the public.

## Supplementary Materials

The following supporting information can be downloaded at: https://www.mdpi.com/article/10.3390/jcm13164884/s1. Figure S1: Frequency according to SUA-groups; Table S1: Definitions and criteria of variables and clinical conditions; Table S2: Clinical characteristics of study population; Table S3: CKM factors and medical conditions in populations with and without hyperu-ricaemia according to HU-7/7 and HU-7/6 diagnostic criteria; Table S4: CKM factors and medical conditions in populations with HU-6 and HU-7; Table S5: Multivariate analysis of CKM factors and medical conditions for HU-7/7 and HU-7/6.

## Abbreviations

AF	atrial fibrillation
CKD	chronic kidney disease
CKM	cardiovascular–kidney–metabolic
CVD	cardiovascular disease
CVR	cardiovascular risk
DM	diabetes mellitus
eGFR	estimated glomerular filtration rate
FLI	fatty liver index
HF	heart failure
HTG	hypertriglyceridaemia
HTN	arterial hypertension
HU	hyperuricaemia
HU-6	HU with SUA ≥ 6 mg/dL
HU-7	HU with SUA ≥ 7 mg/dL
HU-7/6	HU with SUA ≥ 7 mg/dL for men and ≥ 6.0 mg/dL for women
HU-7/7	HU with SUA ≥ 7 mg/dL for both men and women
LDL-C	low-density lipoprotein cholesterol
MetS	metabolic syndrome
MSU	monosodium urate
SLD	steatotic liver disease
SUA	serum uric acid
ULT	urate-lowering drug therapy
WtHR	waist-to-height ratio
