# Supplementary material for: Hyperuricaemia Prevalence Rates According to Their Physiochemical and Epidemiological Diagnostic Criteria and Their Associations with Cardio-Renal-Metabolic Factors: SIMETAP-HU Study
Source: J Clin Med. 2024 Aug 19;13(16):4884. doi: 10.3390/jcm13164884 (PMC11355702; doi:10.3390/jcm13164884)
Supplement: Supplementary file 1 [file jcm-13-04884-s001.zip › jcm-3134786-supplementary.pdf]

Journal of
Clinical Medicine

MDPI

Article

Hyperuricaemia prevalence rates according to their physiochemical and epidemiological diagnostic criteria and their associations with cardio-renal-metabolic factors. SIMETAP-HU Study.

Supplementary Materials

Summary

Table S1: Definitions and criteria of variables and clinical conditions

Table S2: Clinical characteristics of study population

Table S3. CKM factors and medical conditions in populations with and without hyperuricaemia according to HU-7/7 and HU-7/6 diagnostic criteria

Table S4: CKM factors and medical conditions in populations with HU-6 and HU-7

Table S5. Multivariate analysis of CKM factors and medical conditions for HU-7/7 and HU-7/6

Figure S1: Frequency according to SUA-groups

Table S1. Definitions and criteria of variables and clinical conditions

**Morbidities, variables or clinical conditions**	**Criteria and concepts**
Current smoking	Any amount of tobacco during in the past year.
Alcoholism	> 21 standard drink units (SDU) of alcohol consumption (1 SDU= 10 g) per week (male), > 14 SDU per week (female).
Physical inactivity [1]	Moderate-intensity physical activity (e.g., brisk walking) < 150 minutes a week, or vigorous-intensity physical activity (e.g., jogging) < 75 minutes a week (or less of equivalent combination of both), or muscle strengthening exercises < 2 days a week.
Overweight [2]	Body mass index (BMI) 25.0–29.9 kg/m^2 (International Classification of Diseases, 10th Revision, Clinical Modification [ICD-10-CM]: E66.3; International Classification of Primary Care, 2nd edition [ICPC-2]: T83)*
Obesity [2]	BMI ≥ 30 kg/m^2 (ICD-10-CM: E66.9; ICPC-2: T82)*
Abdominal obesity [3]	Increased waist circumference (WC) (≥ 102 cm [men]; ≥ 88 cm [women]) determined with the subject standing using a flexible tape measure adjusted without compressing the skin, at the end of a normal expiration, locating the upper edge of the iliac crests and above that point surrounding the waist parallel to the floor.

Adiposity CUN-BAE [4]	Adiposity or body fat index CUN-BAE (according to its acronym in Spanish, *Clínica Universitaria de Navarra* - Body Adiposity Estimator):<br>• Male: −44.988 + (0.503 x age) + (3.172 x BMI) − (0.026 x BMI^2) − (0.02 x BMI x age) + (0.00021 x BMI^2 x age)<br>• Female: −44.988 + (0.503 x age) + 10.689 + (3.172 x BMI) − (0.026 x BMI^2) + (0.181 x BMI) − (0.02 x BMI x age) − (0.005 x BMI^2) + (0.00021 x BMI^2 x age)
Excess adiposity CUN-BAE [4]	Adiposity or body fat index CUN-BAE > 25% for men or > 35% for female
High waist-to-height ratio (WtHR) [5]	Waist circumference / height ≥ 0.55
Arterial hypertension (HTN) [6]	Systolic blood pressure (SBP) ≥ 140 mmHg and/or diastolic blood pressure (DBP) ≥ 90 mmHg, using the average of two or more readings obtained on two or more occasions, or being on blood pressure-lowering drug therapy (ICD-10-CM: I10, I15; ICPC-2: K86, K87)*
Pulse pressure	SBP – DBP (mmHg)
Diabetes mellitus (DM) [7]	According to the American Diabetes Association (ADA) criteria: fasting plasma glucose (FPG) ≥ 126 mg/dL (7.0 mmol/L) or glycated haemoglobin A1c (HbA_{1c}) ≥ 6.5 % (in International Federation of Clinical Chemistry and Laboratory Medicine [IFCC] units) (≥ 48 mmol/mol) or plasma glucose ≥ 200 mg/dL (11.1 mmol/L) at any time or with oral glucose tolerance test (ICD-10-CM: E10, E11; ICPC-2: T89, T90)*<br>To convert from mg/dL to mmol/L, multiply by 0.05556<br>To convert from % (DCCT) to mmol/mol (IFCC), subtract 2.15 and multiply by 10.929
Prediabetes [7]	According to the ADA criteria: FPG between 100 and 125 mg/dL (5.6–6.9 mmol/L) or HbA_{1c} between 5.7 % and 6.4 % (39–47 mmol/mol) (ICD-10-CM: R73.09; ICPC-2: A91)*
Estimated average glucose (eAG)	28.7 x HbA_{1c} – 46.7 (mg/dL)<br>To convert from mg/dL to mmol/L, multiply by 0.05556
Hypercholesterolaemia	Fasting plasma total cholesterol (TC) concentration ≥ 200 mg/dL (≥ 5.17 mmol/L) (ICD-10-CM: E78; ICPC-2: T93)*<br>To convert from mg/dL to mmol/L, multiply by 0.02586
Hypertriglyceridaemia (HTG)	Fasting plasma triglycerides (TG) concentration ≥ 150 mg/dL (≥ 1.69 mmol/L) (ICD-10-CM: E78; ICPC-2: T93)*<br>To convert from mg/dL to mmol/L, multiply by 0.01129
Low high-density lipoprotein cholesterol (HDL-C)	HDL-C < 40 mg/dL (< 1.03 mmol/L) (male)<br>HDL-C < 50 mg/dL (< 1.29 mmol/L) (female)<br>To convert from mg/dL to mmol/L, multiply by 0.02586
Atherogenic dyslipidaemia	HTG and low HDL-C

Non-high-density lipoprotein cholesterol (Non-HDL-C)	TC – HDL-C
Low-density lipoprotein cholesterol (LDL-C)	TC – HDL-C – (TG/5) mg/dL (not valid for patients with TG > 400 mg/dL)<br>TC – HDL-C – (TG/2.2) mmol/L (not valid for patients with TG > 4.51 mmol/L)
Residual cholesterol (RC)	Very low-density lipoproteins cholesterol (VLDL-C) and remnants.<br>RC= TC – HDL-C – LDL-C
Triglyceride-glucose (TyG) index	Ln (TG x FPG/2)
Atherogenic index of plasma (AIP)	log TG/HDL-C
Fatty liver index (FLI) [8]	$FLI = (e^{0.953*\log_e(TG) + 0.139 \times BMI + 0.718 \times \log_e(GGT) + 0.053 \times WC - 15.745}) / (1 + e^{0.953*\log_e(TG) + 0.139 \times BMI + 0.718 \times \log_e(GGT) + 0.053 \times WC - 15.745}) \times 100$<br>BMI: body mass index; GGT: gamma-glutamyl-transferase; $\log_e$: natural logarithm; TG: triglycerides concentration; WC: waist circumference<br>A FLI 0-30 can be used to rule out steatotic liver disease (SLD) (sensitivity: 87%; negative likelihood ratio: 0.2) and a FLI 60-100 to rule in SLD (specificity 86%; positive likelihood ratio: 4.3)
Metabolic syndrome (MetS) [3]	According to Harmonized Consensus of International Diabetes Federation task force on Epidemiology and Prevention, National Heart, Lung, and Blood Institute, American Heart Association, World Heart Federation, International Atherosclerosis Society, and International Association for the Study of Obesity (3).<br>At least, three of following factors for the European population:<br>• Increased waist circumference (≥ 102 cm [male]; ≥ 88 cm [female])<br>• FPG ≥ 100 mg/dL (≥ 5.6 mmol/L)<br>• HTG (≥ 150 mg/dL [≥ 1.7 mmol/L])<br>• Low HDL-C (< 40 mg/dL [< 1.03 mmol/L] male; < 50 mg/dL [< 1.29 mmol/L] female)<br>• SBP ≥ 130 mmHg or DBP ≥ 85 mmHg or antihypertensive treatment
Hyperuricaemia (HU-7/7) [9-14]	Serum uric acid (SUA) levels ≥ 7.0 mg/dL (416 µmol/L) (HU-7) for both adult males and females, or on urate-lowering drug therapy (ULT). (ICD-10-CM: E79; ICPC-2: T92)*<br>To convert from mg/dL to mmol/L, multiply by 0.05948
Hyperuricaemia (HU-7/6) [15,16]	SUA levels ≥ 7.0 mg/dL (416 µmol/L) (HU-7) for men and ≥ 6.0 mg/dL (357 µmol/L) (HU-6) for women, or on ULT. (ICD-10-CM: E79; ICPC-2: T92)*<br>To convert from mg/dL to mmol/L, multiply by 0.05948
Coronary heart disease (CHD)	Ischemic heart disease, acute myocardial infarction, acute coronary syndrome, coronary revascularization (ICD-10-CM: I20-I25; ICPC-2: K74, K75, K76)*
Cerebrovascular disease (stroke)	Cerebral ischemia, intracranial haemorrhage, transient ischemic attack (ICD-10-CM: I60-I66, I66, I67; ICPC-2: K89, K90K K91)*

Peripheral arterial disease (PAD)	Intermittent claudication, ankle-brachial index ≤ 0.9 (ICD-10-CM: I70.2, I73.9; ICPC-2: K92)*
Atherosclerotic cardiovascular disease (ASCVD)	CHD, stroke, PAD (ICD-10-CM: I70)*
Heart failure (HF) [17-19]	Record of HF diagnosis (ICD-10-CM: I50; ICPC-2: K77)* in the patient's medical record, without differentiating by phenotype based on measurement of left ventricular ejection fraction or based on severity of symptoms and physical activity.
Atrial fibrillation (AF) [20,21]	Record of AF diagnosis (ICD-10-CM: I48; ICPC-2: K78)* in the patient's medical record, without differentiating by phenotypes based on paroxysmal, persistent, long-standing persistent, or permanent AF.
Cardiovascular diseases (CVD)	CHD, stroke, PAD, HF, AF
Estimated glomerular filtration rate (eGFR) [22]	According to Chronic Kidney Disease Epidemiology Collaboration (CKD-EPI) equations:<br>Women with creatinine ≤ 0.7 mg/dL= 144 x $(\text{creatinine})^{-0,329}$ x $(0.993)^{\text{age}}$ mL/min/1.73 m^2 of body surface area<br>Women with creatinine > 0.7 mg/dL= 144 x $(\text{creatinine})^{-1,209}$ x $(0.993)^{\text{age}}$ mL/min/1.73 m^2 of body surface area<br>Men with creatinine ≤ 0.9 mg/dL= 141 x $(\text{creatinine})^{-0,411}$ x $(0.993)^{\text{age}}$ mL/min/1.73 m^2 of body surface area<br>Men with creatinine > 0.9 mg/dL= 141 x $(\text{creatinine})^{-1,209}$ x $(0.993)^{\text{age}}$ mL/min/1.73 m^2 of body surface area
Low eGFR [23]	eGFR < 60 mL/min/1.73 m^2 of body surface area according to CKD-EPI.<br>Not includes:<br>• Stage G1: ≥ 90 mL/min/1.73 m^2<br>• Stage G2: 60 to 89 mL/min/1.73 m^2<br>Includes:<br>• Stage G3a: 45 to 59 mL/min/1.73 m^2<br>• Stage G3b: 30 to 44 mL/min/1.73 m^2<br>• Stage G4: 15 to 29 mL/min/1.73 m^2<br>• Stage G5: < 15 mL/min/1.73 m^2

Albuminuria [23]	Urine albumin-creatinine ratio (uACR) ≥ 30 mg/g (including proteinuria [uACR > 300 mg/g]) (ICD-10-CM: R80; ICPC-2: U98)*<br>Includes:<br>• Stage A2: 30 mg/g to 300 mg/g<br>• Stage A3: > 300 mg/g (proteinuria)<br>Not includes:<br>• Stage A1: < 30 mg/g<br>To convert from mg/g to mg/mmol, multiply by 0.01131
Chronic kidney disease (CKD) [23]	Low eGFR and/or albuminuria (ICD-10-CM: N18; ICPC-2: U99)*
CKD risk [23]	Risk for all-cause mortality, cardiovascular mortality, kidney failure treated with dialysis or transplant, acute kidney failure and progression of kidney disease.<br>• Low risk CKD: G1A1, G2A1<br>• Moderate risk CKD: G1A2, G2A2, G3aA1<br>• High risk CKD: G1A3, G2A3, G3aA2, G3bA1<br>• Very high risk CKD: G3aA3, G3bA2, G3bA3, G4(A1,A2,A3), G5(A1,A2,A3)
Cardiovascular-kidney-metabolic (CKM) syndrome [24]	Systemic disorder attributable to pathophysiological interactions among metabolic risk factors, CKD, and CVD, that includes both individuals at risk for CVD, CKD, and those with existing clinical CVD.<br>• Stage 0: BMI <25 kg/m^2, normal waist circumference (<88 in women and <102 cm in men) without criteria for the other stages.<br>• Stage 1: excess adiposity CUN-BAE, overweight, obesity, abdominal obesity, or prediabetes.<br>• Stage 2: HTN, metabolic risk factors (HTG, DM, MetS), moderate or high risk CKD.<br>• Stage 3: subclinical target organ damage, risk equivalents (high cardiovascular risk [CVR] or very high-risk CKD) among individuals with stages 1 or 2.<br>• Stage 4: clinical CVD including CHD, stroke, PAD, HF, and AF among individuals with stages 1 or 2 (stage 4a: without CKD; stage 4b: with CKD).<br>Stages 3 or 4 are defined as advanced stages of CKM syndrome because they identify individuals at high risk for CVD.
Cardiovascular risk (CVR) categories [25]	Low, moderate, high and very high CVR was estimated for patients from low-risk European countries according to 2021 ESC Guidelines on cardiovascular disease prevention in clinical practice.

** National Center for Health Statistics (NCHS). International Classification of Diseases, 10th Revision, Clinical Modification (ICD-10-CM). [Accessed July 10, 2024]. Available from: https://www.cdc.gov/nchs/icd/icd-10-cm.htm#print*

** World Health Organization. (2009). International Classification of Primary Care, -ICPC-2. [Accessed July 10, 2024]. Available from: https://www.who.int/standards/classifications/other-classifications/international-classification-of-primary-care*

References

1. World Health Organization. WHO Guidelines on Physical Activity and Sedentary Behaviour. 2020. Available online: https://iris.who.int/handle/10665/336656 (accessed on 10 July 2024).
2. *Obesity: Preventing and Managing the Global Epidemic: Report of a WHO Consultation*; WHO technical report series; WHO Consultation on Obesity & World Health Organization: Geneva, Switzerland, 2020; p. 894. Available online: https://apps.who.int/iris/handle/10665/42330 (accessed on 10 July 2024).
3. Alberti, K.G.M.M.; Eckel, R.H.; Grundy, S.M.; Zimmet, P.Z.; Cleeman, J.I.; Donato, K.A.; Fruchart, J.C.; James, W.P.T.; Loria, C.M.; Smith, S.C., Jr. Harmonizing the metabolic syndrome: A joint interim statement of the International Diabetes Federation task force on Epidemiology and Prevention; National Heart, Lung, and Blood Institute; American Heart Association; World Heart Federation; International Atherosclerosis Society; and International Association for the Study of Obesity. *Circulation* **2009**, *120*, 1640–1645. https://doi.org/10.1161/CIRCULATIONAHA.109.192644.
4. Gómez-Ambrosi, J.; Silva, C.; Catalán, V.; Rodríguez, A.; Galofré, J.C.; Escalada, J.; Valentí, V.; Rotellar, F.; Romero, S.; Ramírez, B.; et al. Clinical usefulness of a new equation for estimating body fat. *Diabetes Care* **2012**, *35*, 383–388. https://doi.org//10.2337/dc11-1334.
5. Romero-Saldaña, M.; Fuentes-Jiménez, F.J.; Vaquero-Abellán, M.; Álvarez-Fernández, C.; Aguilera-López, M.D.; Molina-Recio, G. Predictive capacity and cutoff value of waist-to-height ratio in the incidence of metabolic syndrome. *Clin. Nurs. Res.* **2019**, *28*, 676–691. https://doi.org/10.1177/1054773817740533.
6. Mancia, G.; Kreutz, R.; Brunström, M.; Burnier MGrassi, G.; Januszewicz, A.; Muiesan, M.L.; Tsioufis, K.; Agabiti-Rosei, E.; Eae, A.; Azizi, M.; et al. 2023 ESH Guidelines for the management of arterial hypertension. The Task Force for the management of arterial hypertension of the European Society of Hypertension. Endorsed by the European Renal Association (ERA) and the International Society of Hypertension (ISH). *J. Hypertens.* **2023**, *41*, 1874–2071. https://doi.org/10.1097/HJH.0000000000003480.
7. American Diabetes Association Professional Practice Committee. 2. Diagnosis and classification of diabetes: Standards of Care in Diabetes—2024. *Diabetes Care* **2024**, *47* (Suppl. S1), S20–S42. https://doi.org/10.2337/dc24-S002.
8. Bedogni, G.; Bellentani, S.; Miglioli, L.; Masutti, F.; Passalacqua, M.; Castiglione, A.; Tiribelli, C. The Fatty Liver Index: a simple and accurate predictor of hepatic steatosis in the general population. *BMC Gastroenterol.* **2006**, *6*, 33. https://doi.org/10.1186/1471-230X-6-33.
9. Valsaraj, R.; Singh, A.K.; Gangopadhyay, K.K.; Ghoshdastidar, B.; Goyal, G.; Batin, M.; Mukherjee, D.; Sengupta, U.; Chatterjee, S.; Sengupta, N. Management of asymptomatic hyperuricemia: Integrated Diabetes & Endocrine Academy (IDEA) consensus statement. *Diabetes Metab. Syndr.* **2020**, 14, 93–100. https://doi.org/10.1016/j.dsx.2020.01.007.
10. Yamanaka, H. Japanese Society of Gout and Nucleic Acid Metabolism. Japanese guideline for the management of hyperuricemia and gout: second edition. *Nucleosides Nucleotides Nucleic Acids.* **2011**, *30*, 1018–1029. https://doi.org/10.1080/15257770.2011.596496.
11. Khanna, D.; Fitzgerald, J.D.; Khanna, P.P.; Bae, S.; Singh, M.K.; Neogi, T.; Pillinger, M.H.; Merill, J.; Lee, S.; Prakash, S.; et al. 2012 American College of Rheumatology Guidelines for Management of Gout Part I: systematic nonpharmacologic and pharmacologic therapeutic approaches to hyperuricemia. *Arthritis Care Res.* **2012**, *64*,1431–1446. https://doi.org/10.1002/acr.21772.
12. Manara, M.; Bortoluzzi, A.; Favero, M.; Prevete, I.; Scirè, C.; Bianchi, G.; Borghi, C.; Cimmino, M.A.; D'Avola, G.M.; Desideri, G.; et al. Italian Society of Rheumatology recommendations for the management of gout. *Reumatismo* **2013**, 65, 4–21. https://doi.org/10.4081/reumatismo.2013.4.
13. Spanish Society of Rheumatology (SER). Clinical Practice Guidelines for Management of Gout. Available online: https://www.ser.es/wp-content/uploads/2015/09/GuipClinGot_1140226_EN.pdf (accessed on 10 July 2024).

14. Multidisciplinary Expert Task Force on Hyperuricemia and Related Diseases. Chinese Multidisciplinary Expert Consensus on the diagnosis and treatment of hyperuricemia and related diseases. *Chin. Med. J.* **2017**, 130, 2473–2488. https://doi.org/10.4103/0366-6999.216416.
15. Kellgren, J.H.; Jeffrey, M.R.; Ball, J.; Council for International Organizations of Medical Sciences. *The Epidemiology of Chronic Rheumatism*; Blackwell Scientific Publications: Oxford, UK, 1963. Available online: https://ci.nii.ac.jp/ncid/BA33063611 (accessed on 10 July 2024).
16. Johnson, R.J.; Bakris, G.L.; Borghi, C.; Chonchol, M.B.; Feldman, D.; Lanaspa, M.A.; Merriman, T.R.; Moe, O.W.; Mount, D.B.; Lozada, L.G.S.; et al. Hyperuricemia, acute and chronic kidney disease, hypertension, and cardiovascular disease: report of a Scientific Workshop Organized by the National Kidney Foundation. *Am. J. Kidney Dis.* **2018**, *71*, 851–865. https://doi.org/10.1053/j.ajkd.2017.12.009.
17. McDonagh, T.A.; Metra, M.; Adamo, M.; Gardner, R.S.; Baumbach, A.; Böhm, M.; Burri, H.; Butler, J.; Čelutkienė, J.; Chioncel, O.; et al. 2021 ESC Guidelines for the diagnosis and treatment of acute and chronic heart failure. *Eur. Heart J.* **2021**, *42*, 3599–3726.
18. Bozkurt, B.; Coats, A.J.; Tsutsui, H.; Abdelhamid, M.; Adamopoulos, S.; Albert, N.; Anker, S.D.; Atherton, J.; Böhm, M.; Butler, J.; et al. Universal definition and classification of heart failure: A report of the Heart Failure Society of America, Heart Failure Association of the European Society of Cardiology, Japanese Heart Failure Society and Writing Committee of the Universal Definition of Heart Failure. *Eur. J. Heart Fail.* **2021**, *23*, 352–380.
19. Heidenreich, P.A.; Bozkurt, B.; Aguilar, D.; Allen, L.A.; Byun, J.J.; Colvin, M.M.; Deswal, A.; Drazner, M.H.; Dunlay, S.M.; Evers, L.R.; et al. 2022 AHA/ACC/HFSA Guideline for the management of heart failure: A Report of the American College of Cardiology/American Heart Association Joint Committee on Clinical Practice Guidelines. *J. Am. Coll. Cardiol.* **2022**, *79*, e263–e421.
20. Hin Hindricks, G.; Potpara, T.; Dagres, N.; Arbelo, E.; Bax, J.J.; Blomström-Lundqvist, C.; Boriani, G.; Castella, M.; Dan, G.A.; Dilaveris, P.E.; et al. 2020 ESC Guidelines for the diagnosis and management of atrial fibrillation developed in collaboration with the European Association for Cardio-Thoracic Surgery (EACTS): The Task Force for the diagnosis and management of atrial fibrillation of the European Society of Cardiology (ESC). Developed with the special contribution of the European Heart Rhythm Association (EHRA) of the ESC. *Eur. Heart J.* **2021**, *42*, 373–498.
21. Joglar, J.A.; Chung, M.K.; Armbruster, A.L.; Benjamin, E.J.; Chyou, J.Y.; Cronin, E.M.; Deswal, A.; Eckhardt, L.L.; Goldberger, Z.D.; Gopinathannair, R.; et al. 2023 ACC/AHA/ACCP/HRS Guideline for the diagnosis and management of atrial fibrillation: A report of the American College of Cardiology/American Heart Association Joint Committee on Clinical Practice Guidelines. *Circulation* **2024**, 149, e1–e156. https://doi.org/10.1161/CIR.0000000000001193.
22. Levey, A.S.; Stevens, L.A.; Schmid, C.H.; Zhang, Y.L.; Castro, A.F., 3rd; Feldman, H.I.; Kusek, J.W.; Eggers, P.; Van Lente, F.; Greene, T.; et al. CKD-EPI (Chronic Kidney Disease Epidemiology Collaboration). A new equation to estimate glomerular filtration rate. *Ann. Intern. Med.* **2009**, 150, 604–12. https://doi.org/10.7326/0003-4819-150-9-200905050-00006.
23. Stevens, P.E.; Ahmed, S.B.; Carrero, J.J.; Foster, B.; Francis, A.; Hall, R.K.; Herrington, W.G.; Hill, G.; Inker, L.A.; Kazancıoğlu, R.; et al. Kidney Disease: Improving Global Outcomes (KDIGO) CKD Work Group. KDIGO 2024 Clinical practice guideline for the evaluation and management of chronic kidney disease. *Kidney Int.* **2024**, *105*, S117–S314. https://doi.org/10.1016/j.kint.2023.10.018.
24. Ndumele, C.E.; Rangaswami, J.; Chow, S.L.; Neeland, I.J.; Tuttle, K.R.; Khan, S.S.; Coresh, J.; Mathew, R.O.; Baker-Smith, C.M.; Carnethon, M.R.; et al. American Heart Association. Cardiovascular-kidney-metabolic health: A Presidential Advisory from the American Heart Association. *Circulation* **2023**, 148, 1606–1635. https://doi.org/10.1161/CIR.0000000000001184.
25. Visseren, F.L.J.; Mach, F.; Smulders, Y.M.; Carballo, D.; Koskinas, K.C.; Bäck, M.; Benetos, A.; Biffi, A.; Boavida, J.-M.; Capodanno, D.; et al. ESC Scientific Document Group. 2021 ESC Guidelines on cardiovascular disease prevention in clinical

practice. Developed by the Task Force for cardiovascular disease prevention in clinical practice with representatives of the European Society of Cardiology and 12 medical societies. With the special contribution of the European Association of Preventive Cardiology (EAPC). *Eur. Heart J.* **2021**, *42*, 3227–3337. https://doi.org/10.1093/eurheartj/ehab484.

Table S2. Clinical characteristics of study population.

	Male			Female			Diff. in means	*p*
	No.	Mean	SD	No.	Mean	SD		
Age (yr)	2857	55.3	16.8	3632	55.1	18.0	0.2	0.640
BMI (kg/m^2)	2857	27.9	4.5	3632	27.2	5.6	0.8	< 0.001
WC (cm)	2857	98.0	12.6	3632	89.7	14.1	8.4	< 0.001
WtHR	2857	0.57	0.08	3632	0.57	0.10	0.01	0.001
CUN-BAE-adiposity	2857	28.9	6.3	3632	39.3	7.5	-10.4	< 0.001
SBP (mmHg)	2857	124.1	14.1	3632	120.3	16.2	3.8	< 0.001
DBP (mmHg)	2857	74.9	9.4	3632	72.1	9.9	2.7	< 0.001
Pulse pressure (mmHg)	2857	49.2	11.5	3632	48.1	12.2	1.1	< 0.001
FPG (mg/dL) [a]	2857	99.7	28.3	3632	93.1	23.3	6.6	< 0.001
HbA_{1c} (%) [b]	2327	5.71	0.93	2851	5.57	0.86	0.14	< 0.001
eAG (mg/dL) [a]	2327	117.1	26.7	2851	113.2	24.6	3.9	< 0.001
TC (mg/dL) [c]	2857	188.3	39.1	3632	196.4	39.2	-8.1	< 0.001
HDL-C (mg/dL) [c]	2857	49.2	12.6	3632	59.3	14.7	-10.1	< 0.001
LDL-C (mg/dL) [c]	2813	112.6	34.4	3614	115.5	34.6	-2.9	0.001
RC (mg/dL) [c]	2813	25.2	13.6	3614	21.2	11.0	4.0	< 0.001
Non-HDL-C (mg/dL) [c]	2857	139.0	38.8	3632	137.1	38.2	1.9	0.047
TG (mg/dL) [d]	2857	135.9	101.2	3632	108.5	64.0	27.4	< 0.001
Non-HDL-C/HDL-C	2857	3.04	1.25	3632	2.47	0.97	0.56	< 0.001
LDL-C/HDL-C	2813	2.42	0.97	3614	2.06	0.79	0.36	< 0.001
TG / HDL-C	2857	3.14	3.26	3632	2.05	1.70	1.09	< 0.001
AIP	2857	0.03	0.29	3632	-0.14	0.27	0.17	< 0.001
TyG index	2857	8.63	0.61	3632	8.38	0.58	0.26	< 0.001
SUA (mg/dL) [e]	2857	5.70	1.43	3632	4.39	1.25	1.31	< 0.001
AST (U/L)	2112	25.6	47.1	2700	21.0	39.7	4.6	< 0.001
ALT (U/L)	2809	29.2	19.1	3546	21.5	14.1	7.7	< 0.001
GGT (U/L)	2673	42.1	64.4	3395	26.4	34.2	15.7	< 0.001
FLI (0-100)	2673	54.6	28.1	3395	37.7	30.3	16.9	< 0.001
Creatinine (mg/dL) [f]	2857	0.96	0.30	3632	0.75	0.26	0.2	< 0.001
eGFR (mL/min/1.73 m^2)	2857	89.7	19.7	3632	91.1	21.0	-1.4	0.006
uACR (mg/g) [g]	2857	20.7	79.9	3632	13.1	39.4	7.6	< 0.001

AIP: atherogenic index of plasma; ALT: alanine aminotransferase; AST: aspartate aminotransferase; BMI: body mass index; CUN-BAE-adiposity: adiposity or body fat index CUN-BAE (according to its acronym in Spanish, Clínica Universitaria de Navarra - Body Adiposity Estimator); DBP: diastolic blood pressure; eAG: estimated average glucose; eGFR: estimated glomerular filtration rate; FGP: fasting plasma glucose; FLI: fatty liver index; GGT: gamma-glutamyl transferase; HbA_{1c}: glycated haemoglobin A_{1c}; HDL-C: high-density lipoprotein

cholesterol; LDL-C: low-density lipoprotein cholesterol; RC: residual cholesterol (very low-density lipoproteins cholesterol and remnants); SBP: systolic blood pressure; SUA: serum uric acid; TC: total cholesterol; TG: triglycerides; TyG index: triglyceride and glucose index; uACR: urine albumin-creatinine ratio; WC: waist circumference; WtHR: waist-to-height ratio. The definitions of the variables are shown in Table S1 (Suppl. Material).

p: p-value of the difference in means

[a] *To convert from mg/dL to mmol/L, multiply by 0.05556*

[b] *To convert from % (DCCT) to mmol/mol (IFCC), subtract 2.15 and multiply by 10.929*

[c] *To convert from mg/dL to mmol/L, multiply by 0.02586*

[d] *To convert from mg/dL to mmol/L, multiply by 0.01129*

[e] *To convert from mg/dL to mmol/L, multiply by 0.05948*

[f] *To convert from mg/dL to mmol/L, multiply by 0.08842*

[g] *To convert from mg/g to mg/mmol, multiply by 0.01131*

Table S3. CKM factors and medical conditions in populations with and without hyperuricaemia according to HU-7/7 and HU-7/6 diagnostic criteria

	With HU-7/7 N= 740 No. (%)	Without HU-7/7 N= 5749 No. (%)	*p*	OR (95% CI)	With HU-7/6 N= 987 No. (%)	Without HU-7/6 N= 5502 No. (%)	*p*	OR (95% CI)
Current smoking	148 (20.0)	1251 (21.8)	0.273	0.9 (0.7–1.1)	173 (17.5)	1226 (22.3)	0.001	0.7 (0.6–0.9)
Alcoholism	133 (18.0)	469 (8.2)	<0.001	2.5 (2.0–3.0)	144 (14.6)	458 (8.3)	<0.001	1.8 (1.5–2.3)
Physical inactivity	365 (49.3)	2660 (46.3)	0.117	1.3 (1.0–1.3)	505 (51.2)	2520 (45.8)	0.002	1.2 (1.1–1.4)
Overweight	311 (42.0)	2169 (37.7)	0.024	1.2 (1.0–1.4)	400 (40.5)	2080 (37.8)	0.105	1.1 (1.0–1.3)
Obesity	321 (43.4)	1481 (25.8)	<0.001	2.2 (1.9–2.6)	447 (45.3)	1355 (24.6)	<0.001	2.5 (2.2–2.9)
Abdominal obesity	438 (59.2)	2440 (42.4)	<0.001	2.0 (1.7–2.3)	634 (64.2)	2244 (40.8)	<0.001	2.6 (2.3–3.0)
High WHtR	575 (77.7)	3064 (53.3)	<0.001	3.1 (2.5–3.7)	773 (78.3)	2866 (52.1)	<0.001	3.3 (2.8–3.9)
Excess adiposity CUN-BAE	665 (89.9)	4090 (71.1)	<0.001	3.6 (2.8–4.6)	901 (91.3)	3854 (70.0)	<0.001	4.5 (3.6–5.6)
Prediabetes	235 (31.8)	1199 (20.9)	<0.001	1.8 (1.5–2.1)	306 (31.0)	1128 (20.5)	<0.001	1.7 (1.5–2.0)
Diabetes	174 (23.5)	843 (14.7)	<0.001	1.8 (1.5–2.2)	244 (24.7)	773 (14.0)	<0.001	2.0 (1.7–2.4)
Hypertension	480 (64.9)	2043 (35.4)	<0.001	3.3 (2.9–3.9)	659 (66.8)	1855 (33.7)	<0.001	3.9 (3.4–4.6)
Hypercholesterol-aemia	531 (71.8)	3515 (61.1)	<0.001	1.6 (1.4–1.9)	716 (72.5)	3330 (60.5)	<0.001	1.7 (1.5–2.0)
Low HDL-C	281 (38.0)	1510 (26.3)	<0.001	1.7 (1.5–2.0)	365 (37.0)	1426 (25.9)	<0.001	1.7 (1.4–1.9)
Hypertriglycer-idaemia	360 (48.6)	1566 (27.2)	<0.001	2.5 (2.2–3.0)	462 (46.8)	1464 (26.6)	<0.001	2.4 (2.1–2.8)
Metabolic syn-drome	493 (66.6)	2321 (40.4)	<0.001	2.9 (2.5–3.5)	681 (69.0)	2133 (38.8)	<0.001	3.5 (3.0–4.1)
FLI ≥ 60 [a,b]	454 (66.5)	1683 (31.3)	<0.001	4.4 (3.7–5.2)	580 (63.3)	1557 (30.2)	<0.001	4.0 (3.4–4.6)
ASCVD	117 (15.8)	488 (8.5)	<0.001	2.0 (1.6–2.5)	159 (16.1)	446 (8.1)	<0.001	2.2 (1.8–2.6)
CHD	60 (8.1)	255 (4.4)	<0.001	1.9 (1.4–2.5)	78 (7.9)	237 (4.3)	<0.001	1.9 (1.5–2.5)
Stroke	46 (6.2)	201 (3.5)	<0.001	1.8 (1.3–2.5)	67 (6.8)	180 (3.3)	<0.001	2.2 (1.6–2.9)
PAD	33 (4.5)	115 (2.0)	<0.001	2.3 (1.5–3.4)	42 (4.3)	106 (1.9)	<0.001	2.3 (1.6–3.3)

Erectile dysfunction [c,d]	122 (21.0)	377 (16.6)	0.012	1.3 (1.1–1.7)	122 (21.0)	377 (16.6)	0.012	1.3 (1.1–1.7)
Heart failure	46 (6.2)	135 (2.3)	<0.001	2.8 (2.0–3.9)	65 (6.6)	116 (2.1)	<0.001	3.3 (2.4–4.5)
Atrial fibrillation	49 (6.6)	196 (3.4)	<0.001	2.0 (1.5–2.8)	74 (7.5)	171 (3.1)	<0.001	2.5 (1.9–3.3)
Albuminuria	103 (13.9)	287 (5.0)	<0.001	3.1 (2.4–3.9)	129 (13.1)	261 (4.7)	<0.001	3.0 (2.4–3.8)
Low eGFR	157 (21.2)	357 (6.2)	<0.001	4.1 (3.3–5.0)	226 (22.9)	288 (6.2)	<0.001	5.4 (4.4–6.5)
CKD	196 (26.5)	548 (9.5)	<0.001	3.4 (2.8–4.1)	274 (27.8)	470 (8.5)	<0.001	4.1 (3.5–4.9)
GLT	139 (18.8)	692 (12.0)	<0.001	1.7 (1.4–2.1)	193 (19.6)	638 (11.6)	<0.001	1.9 (1.6–2.2)
BPLT	458 (61.9)	1846 (32.1)	<0.001	3.4 (2.9–4.0)	630 (63.8)	1674 (30.4)	<0.001	4.0 (3.5–4.7)
LLT	302 (40.8)	1528 (26.6)	<0.001	1.9 (1.6–2.2)	418 (42.4)	1412 (25.7)	<0.001	2.1 (1.8–2.4)
Low CVR	86 (11.6)	2019 (35.1)	<0.001	0.2 (0.2–0.3)	111 (11.2)	1994 (36.2)	<0.001	0.2 (0.2–0.3)
Moderate CVR	162 (21.9)	1203 (20.9)	0.543	1.1 (0.9–1.3)	203 (20.6)	1162 (21.1)	0.695	1.0 (0.8–1.1)
High CVR	149 (20.1)	864 (15.0)	<0.001	1.4 (1.2–1.7)	200 (20.3)	813 (14.8)	<0.001	1.5 (1.2–1.7)
Very high CVR	343 (46.4)	1653 (28.8)	<0.001	2.1 (1.8–2.5)	473 (47.9)	2006 (36.5)	<0.001	1.6 (1.4–1.8)

HU-7/7: hyperuricaemia with serum uric acid (SUA) ≥7.0 mg/dL (416 µmol /L) for both men and women; HU-7/6: SUA ≥7.0 mg/dL (416 µmol /L) for men and ≥6.0 mg/dL (357 µmol/L) for women; No. (%): cases number (percentage); OR: odds ratio; CI: confidence interval; *p*: p-value of the difference in percentage; [a] N= 683 (with HU-7/7), 5385 (without HU-7/7); [b] N= 916 (with HU-7/6), 5152 (without HU-7/6); [c] N= 581 (with HU-7/7), 2276 (without HU-7/7); [d] N= 581 (with HU-7/6), 2276 (without HU-7/6)

ASCVD: atherosclerotic cardiovascular disease; BPLT: blood pressure-lowering drug therapy; CHD: coronary heart disease; CKD: chronic kidney disease; CKM: cardiovascular-kidney-metabolic; CUN-BAE: according to its acronym in Spanish, *Clínica Universitaria de Navarra* - Body Adiposity Estimator; eGFR: estimated glomerular filtration rate; FLI: fatty liver index; GLT: glycaemic-lowering drug therapy; HDL-C: high-density lipoprotein cholesterol; LLT: lipid-lowering drug therapy; PAD: peripheral arterial disease; CVR: cardiovascular risk; WHtR: waist-to-height ratio. The definitions of the CKM factors, comorbidities or medical conditions are shown in Table S1 (Suppl. Material).

Table S4. CKM factors and medical conditions in populations with HU-6 and HU-7

	HU-6	HU-7		HU-6 (F) *vs.* HU-7 (M)		HU-7 (F) *vs.* HU-7 (M)	
	Female (F) N= 406 No. (%)	Female (F) N= 159 No. (%)	Male (M) N= 581 No. (%)	*p*	OR (95% CI)	*p*	OR (95% CI)
Current smoking	45 (11.1)	20 (12.6)	128 (22.0)	<0.001	0.4 (0.3; 0.6)	0.008	0.5 (0.3; 0.8)
Alcoholism	22 (5.4)	11 (6.9)	122 (21.0)	<0.001	0.2 (0.1; 0.3)	<0.001	0.3 (0.1; 0.5)
Physical inactivity	237 (59.3)	97 (61.0)	268 (46.1)	<0.001	1.6 (1.3; 2.1)	0.001	1.8 (1.3; 2.6)
Overweight	141 (34.7)	52 (32.7)	259 (44.6)	0.002	0.7 (0.5; 0.9)	0.007	0.6 (0.4; 0.9)
Obesity	211 (52.0)	85 (53.5)	236 (40.6)	<0.001	1.6 (1.2; 2.0)	0.004	1.7 (1.2; 2.4)
Abdominal obesity	323 (79.6)	127 (79.9)	311 (53.5)	<0.001	3.4 (2.5; 4.5)	<0.001	3.4 (2.3; 5.2)
High WtHR	331 (81.5)	133 (83.6)	442 (76.1)	0.041	1.4 (1.0; 1.9)	0.042	1.6 (1.0; 2.6)
Excess adiposity CUN-BAE	384 (94.6)	148 (93.1)	517 (89.0)	<0.001	2.2 (1.3; 3.6)	0.129	1.7 (0.9; 3.2)
Prediabetes	119 (29.3)	48 (30.2)	187 (32.2)	0.336	0.9 (0.7; 1.2)	0.631	0.9 (0.6; 1.3)
Diabetes	120 (29.6)	50 (31.4)	124 (21.3)	0.003	1.5 (1.2; 2.1)	0.008	1.7 (1.1; 2.5)
Hypertension	304 (74.9)	125 (78.6)	355 (61.1)	<0.001	1.9 (1.4; 2.5)	<0.001	2.3 (1.5; 3.5)
Hypercholesterolaemia	314 (77.3)	129 (81.1)	402 (69.2)	<0.001	1.5 (1.1; 2.0)	0.003	1.9 (1.2; 3.0)
Low HDL-C	154 (37.9)	70 (44.0)	211 (36.3)	0.606	1.1 (0.8; 1.4)	0.076	1.4 (1.0; 2.0)
HTG	181 (44.6)	79 (49.7)	281 (48.4)	0.242	0.9 (0.7; 1.1)	0.766	1.1 (0.7; 1.5)

MetS	313 (77.1)	125 (78.6)	368 (63.3)	<0.001	1.9 (1.5; 2.6)	<0.001	2.1 (1.4; 3.2)
FLI ≥ 60 *	219 (57.5)	93 (62.8)	361 (67.5)	0.002	0.7 (0.5; 0.9)	0.290	0.8 (0.6; 1.2)
ASCVD	67 (16.5)	25 (15.7)	92 (15.8)	0.778	1.1 (0.7; 1.5)	0.973	1.0 (0.6; 1.6)
CHD	30 (7.4)	12 (7.6)	48 (8.3)	0.618	0.9 (0.6; 1.4)	0.771	0.9 (0.5; 1.8)
Stroke	33 (8.1)	12 (7.6)	34 (5.9)	0.161	1.4 (0.9; 2.3)	0.431	1.3 (0.7; 2.6)
PAD	16 (3.9)	7 (4.4)	26 (4.5)	0.679	0.9 (0.5; 1.7)	0.966	1.0 (0.4; 2.3)
Heart failure	35 (8.6)	16 (10.1)	30 (5.2)	0.031	1.7 (1.0; 2.9)	0.023	2.1 (1.1; 3.9)
Atrial fibrillation	44 (10.8)	19 (11.9)	30 (5.2)	<0.001	2.2 (1.4; 3.6)	0.002	2.5 (1.4; 4.6)
Albuminuria	55 (13.5)	29 (18.2)	74 (12.7)	0.710	1.1 (0.7; 1.6)	0.076	1.5 (1.0; 2.4)
Low eGFR	129 (31.8)	60 (37.7)	97 (16.7)	<0.001	2.3 (1.7; 3.1)	<0.001	3.0 (2.1; 4.5)
CKD	146 (36.0)	68 (42.8)	128 (22.0)	<0.001	2.0 (1.5; 2.6)	<0.001	2.6 (1.8; 3.8)
GLT	100 (24.6)	46 (28.9)	93 (16.0)	<0.001	1.7 (1.2; 2.4)	<0.001	2.1 (1.4; 3.2)
BPLT	296 (72.9)	124 (78.0)	334 (57.5)	<0.001	2.0 (1.5; 2.6)	<0.001	2.6 (1.7; 3.9)
LLT	190 (46.8)	74 (46.5)	228 (39.2)	0.018	1.4 (1.1; 1.8)	0.097	1.3 (0.9; 1.9)
ULT	21 (5.2)	21 (13.2)	116 (20.0)	<0.001	0.2 (0.1; 0.4)	0.052	0.6 (0.4; 1.0)
Low CVR	34 (8.4)	9 (5.7)	77 (13.3)	0.017	0.6 (0.4; 0.9)	0.008	0.4 (0.2; 0.8)
Moderate CVR	67 (16.5)	26 (16.4)	136 (23.4)	0.008	0.6 (0.5; 0.9)	0.056	0.6 (0.4; 1.0)
High CVR	82 (20.2)	31 (19.5)	118 (20.3)	0.967	1.0 (0.7; 1.4)	0.821	1.0 (0.6; 1.5)
Very high CVR	223 (54.9)	93 (58.5)	250 (43.0)	<0.001	1.6 (1.2; 2.1)	<0.001	1.9 (1.3; 2.7)

HU-7: serum uric acid (SUA) ≥ 7.0 mg/dL (416 µmol /L); HU-6: SUA ≥ 6.0 mg/dL (357 µmol/L); No. (%): cases number (percentage); *p*: *p*-value of the difference in percentage; OR: odds ratio; CI: confidence interval; * N (FLI)= 535 (M with HU-7), 148 (F with HU-7); 381 (F with HU-6)

ASCVD: atherosclerotic cardiovascular disease; BPLT: blood pressure-lowering drug therapy; CHD: coronary heart disease; CKD: chronic kidney disease; CKM: cardiovascular-kidney-metabolic; CUN-BAE: according to its acronym in Spanish, *Clínica Universitaria de Navarra* - Body Adiposity Estimator; CVR: cardiovascular risk; eGFR: estimated glomerular filtration rate; FLI: fatty liver index; GLT: glycaemic-lowering drug therapy; HDL-C: high-density lipoprotein cholesterol; HTG: hypertriglyceridaemia; LLT: lipid-lowering drug therapy; MetS: metabolic syndrome; PAD: peripheral arterial disease; ULT: urate-lowering drug therapy; WtHR: waist-to-height ratio. The definitions of the cardiovascular-kidney-metabolic factors, comorbidities or medical conditions are shown in Table S1 (Suppl. Material).

Table S5. Multivariate analysis of CKM factors and medical conditions for HU-7/7 and HU-7/6

Overall population	HU-7/7*				HU-7/6*			
	Wald	β [a]	OR Exp(β) [b]	*p* [c]	Wald	β [a]	OR Exp(β) [b]	*p* [c]
Low eGFR	59.0	0.91 (0.12)	2.48 (1.97–3.13)	<0.001	126.8	1.23 (0.13)	3.41 (2.75–4.22)	<0.001
Hypertension	56.3	0.69 (0.09)	2.00 (1.67–2.39)	<0.001	109.3	0.85 (0.08)	2.34 (2.00–2.74)	<0.001
Alcoholism	60.3	0.89 (0.11)	2.43 (1.94–3.03)	<0.001	33.4	0.64 (0.11)	1.89 (1.52–2.35)	<0.001
HTG	43.1	0.55 (0.08)	1.74 (1.47–2.05)	<0.001	47.1	0.53 (0.08)	1.69 (1.46–1.96)	<0.001
Obesity	18.0	0.66 (0.16)	1.93 (1.42–2.62)	<0.001	83.7	1.02 (0.11)	2.77 (2.23–3.45)	<0.001
Overweight	13.0	0.49 (0.14)	1.64 (1.25–2.15)	<0.001	37.2	0.66 (0.11)	1.94 (1.57–2.40)	<0.001
Albuminuria	10.5	0.44 (0.14)	1.56 (1.19–2.03)	0.001	4.23	0.27 (0.13)	1.31 (1.01–1.68)	0.040
Prediabetes	7.8	0.25 (0.09)	1.29 (1.08–1.54)	0.005				NS
High WtHR	4.2	0.25 (0.12)	1.29 (1.01–1.65)	0.041				NS
**Female population**	**HU-7***				**HU-6***			
	Wald	β [a]	OR Exp(β) [b]	*p* [c]	Wald	β [a]	OR Exp(β) [b]	*p* [c]
Low eGFR	45.7	1.33 (0.20)	3.77 (2.57–5.34)	<0.001	108.0	1.50 (0.14)	4.47 (3.37–5.92)	<0.001
Hypertension	46.5	1.45 (0.21)	4.26 (2.81–6.46)	<0.001	81.4	1.21 (0.14)	3.36 (2.60–4.37)	<0.001
HTG	18.1	0.74 (0.17)	2.10 (1.49–2.95)	<0.001	20.6	0.55 (0.12)	1.73 (1.37–2.19)	<0.001
Alcoholism	13.2	1.30 (0.36)	3.65 (1.82–7.36)	<0.001	14.9	1.08 (0.28)	2.93 (1.70–5.05)	<0.001
Albuminuria	7.2	0.66 (0.25)	1.94 (1.19–3.14)	0.007				NS
Central obesity				NS	24.5	0.78 (0.16)	2.29 (1.61–2.98)	<0.001
Obesity				NS	6.3	0.34 (0.14)	1.40 (1.08–1.83)	0.012

Male population	HU-7*			
	Wald	β [a]	OR Exp(β) [b]	*p* [c]
Low eGFR	47.3	1.06 (0.16)	2.90 (2.14–3.92)	<0.001
Hypertension	41.7	0.66 (0.10)	1.94 (1.59–2.38)	<0.001
Central obesity	29.3	0.54 (0.10)	1.72 (1.41–2.10)	<0.001
HTG	19.2	0.43 (0.10)	1.54 (1.27–1.87)	<0.001
Alcoholism	6.2	0.30 (0.12)	1.36 (1.07–1.72)	0.013

* HU-7/7: hyperuricaemia (HU) with serum uric acid (SUA) ≥7.0 mg/dL (416 µmol/L) (HU-7) for both male and female. HU-7/6: SUA ≥7.0 mg/dL in male and ≥6.0 mg/dL (357 µmol/L) (HU-6) in female. [a] β coefficient (± deviation). [b] Odds-ratio Exp (β) (95% confidence interval). [c] p: p-value of Wald test with one degree of freedom; NS: not significant.

eGFR: estimated glomerular filtration rate; HTG: hypertriglyceridaemia; WtHR: waist-to-height ratio. Definitions of the clinical conditions are shown in Table S1 (Suppl. Material).

Figure S1. Frequency according to SUA-groups

Male

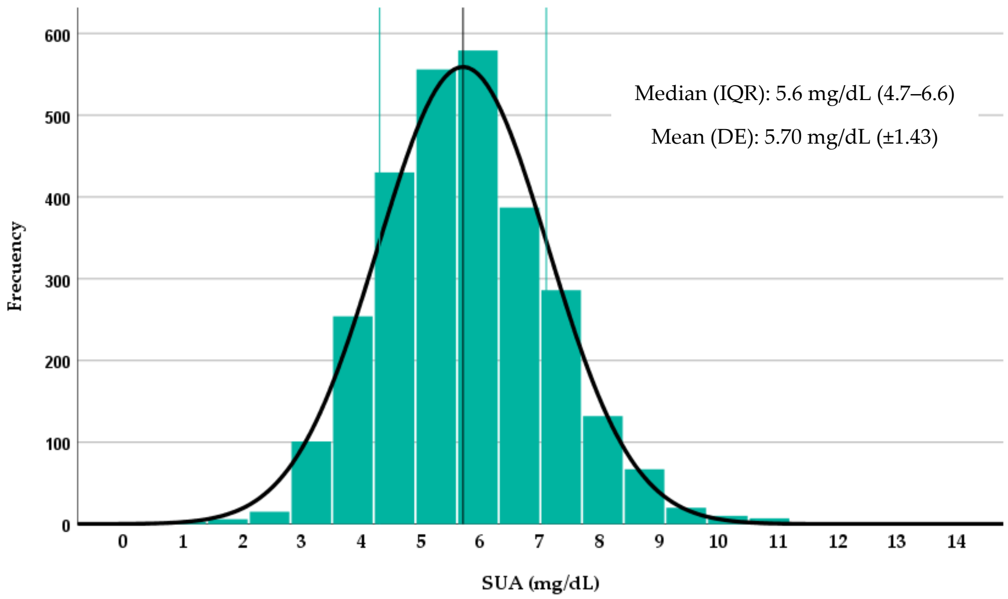

Female

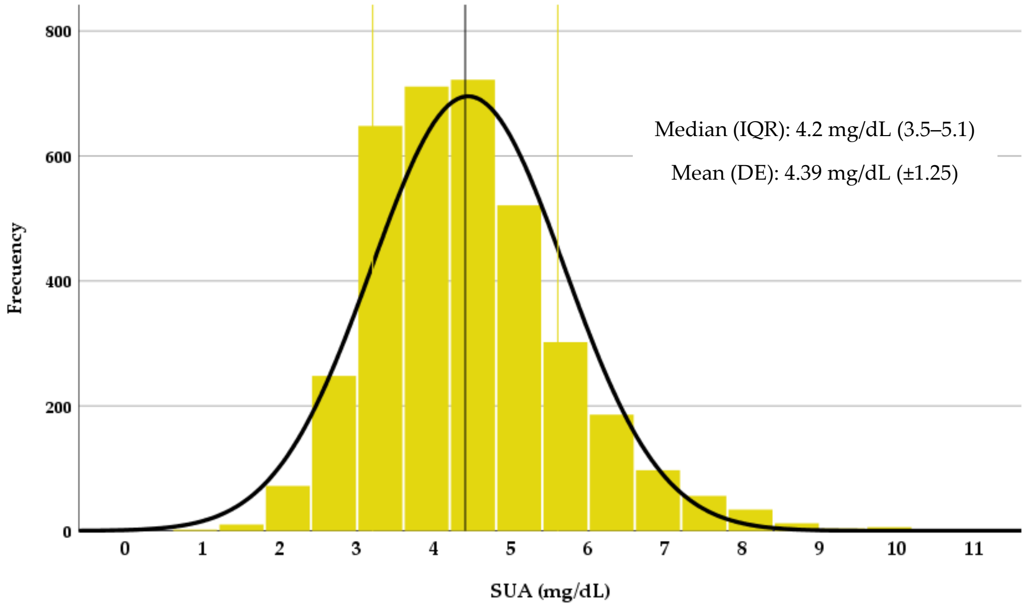

SUA: serum uric acid; IQR: interquartile range; SD: standard deviation
